# Supplementary material for: The Systems Biology Research Tool: evolvable open-source software
Source: BMC Syst Biol. 2008 Jun 29;2:55. doi: 10.1186/1752-0509-2-55 (PMC2446383; doi:10.1186/1752-0509-2-55)
Supplement: Additional file 1 — SBRT Archive. An archive of the current version of the Systems Biology Research Tool. [file 1752-0509-2-55-S1.zip › sbrt-1.4.0/doc/developers_guide/api/sbrt/kernel/math/geom/class-use/ParallelHyperplanes.html]

Uses of Class sbrt.kernel.math.geom.ParallelHyperplanes


|  |  |  |
| --- | --- | --- |
| **Overview** \| **Package** \| **Class** \| **Use** \| **Tree** \| **Deprecated** \| **Index** \| **Help** | |  |
| PREV   NEXT | **FRAMES**    **NO FRAMES**     **All Classes** |  |


---


## **Uses of Class sbrt.kernel.math.geom.ParallelHyperplanes**

| Packages that use ParallelHyperplanes | |
| --- | --- |
| **sbrt.kernel.math.geom** | Provides classes and interfaces for creating objects relevant to computational geometry. |
| **sbrt.shell.mng.geom** | Provides classes and for creating geometry process managers. |

Uses of ParallelHyperplanes in sbrt.kernel.math.geom

| Methods in sbrt.kernel.math.geom that return types with arguments of type ParallelHyperplanes | |
| --- | --- |
| `java.util.Set<ParallelHyperplanes<V>>` | `ConvexPolytope.getBoundingPlanes()`             Returns the set of bounding hyperplanes that define this convex polytope. |
| `UnmodifiableSet<ParallelHyperplanes<V>>` | `HashConvexPolytope.getBoundingPlanes()`             Returns the set of bounding hyperplanes that define this convex polytope. |
| `java.util.Set<ParallelHyperplanes<V>>` | `HashConvexPolytopeOld.getBoundingPlanes()`             Returns the set of bounding hyperplanes that define this convex polytope. |

Constructor parameters in sbrt.kernel.math.geom with type arguments of type ParallelHyperplanes

`HashConvexPolytope(java.util.Set<ParallelHyperplanes<V>> boundingPlanes)`
  
          Constructs a new convex poltyope from the provided linear system solution
and variable bounds.

Uses of ParallelHyperplanes in sbrt.shell.mng.geom

| Methods in sbrt.shell.mng.geom that return types with arguments of type ParallelHyperplanes | |
| --- | --- |
| `static java.util.Set<ParallelHyperplanes<java.lang.String>>` | `CdHarManager.getPlanes(ManagerInput<java.lang.String,java.lang.String> input, java.io.PrintWriter out)`             Returns the list of sets contained in the file with the name contained in the provided input. |
| `static java.util.Set<ParallelHyperplanes<java.lang.String>>` | `CdHarManager.getPlanes(java.lang.String fileName)`             Returns the list of sets contained in the file with the provided name. |
| `static java.util.Set<ParallelHyperplanes<java.lang.String>>` | `CdHarManager.getPlanes(java.lang.String fileName, java.io.PrintWriter out)`             Returns the list of sets contained in the file with the provided name. |

---


|  |  |  |
| --- | --- | --- |
| **Overview** \| **Package** \| **Class** \| **Use** \| **Tree** \| **Deprecated** \| **Index** \| **Help** | |  |
| PREV   NEXT | **FRAMES**    **NO FRAMES**     **All Classes** |  |


---
